# The Diagnostic Value of Metagenomic Next–Generation Sequencing in Lower Respiratory Tract Infection

**DOI:** 10.3389/fcimb.2021.694756

**Published:** 2021-09-09

**Authors:** Yan Zheng, Xiaojian Qiu, Ting Wang, Jie Zhang

**Affiliations:** Department of Respiratory and Critical Care, Beijing Tian Tan Hospital, Capital Medical University, Beijing, China

**Keywords:** metagenome, next-generation sequencing technology, lower respiratory tract infection, sampling methods, diagnostic value

## Abstract

Lower respiratory tract infections are associated with high morbidity and mortality and significant clinical harm. Due to the limited ability of traditional pathogen detection methods, anti-infective therapy is mostly empirical. Therefore, it is difficult to adopt targeted drug therapy. In recent years, metagenomic next-generation sequencing (mNGS) technology has provided a promising means for pathogen-specific diagnosis and updated the diagnostic strategy for lower respiratory tract infections. This article reviews the diagnostic value of mNGS for lower respiratory tract infections, the impact of different sampling methods on the detection efficiency of mNGS, and current technical difficulties in the clinical application of mNGS.

## Introduction

Lower respiratory tract infections are the main cause of death from infectious causes worldwide (Langelier et al., 2018). When the pathogen of a respiratory tract infection is not clearly identified, it is often difficult to make targeted drug treatment. This in turn delays improvement of the patient’s condition and leads to the aggravation or worsening of symptoms, or even death. Some refractory lower respiratory tract infections can be difficult to diagnose and treat. In these cases, it is particularly important to accurately identify the pathogen of the lower respiratory tract infection and make targeted treatment. However, in China, the etiological diagnosis of nearly half of patients with pulmonary infection is not clear (Zhu et al., 2018).

The identification of pathogens in the lower respiratory tract has long relied on traditional microbial culture, antigen and/or antibody immunological methods, and polymerase chain reaction (PCR) detection. The most common types of specimens in traditional microbial culture methods are blood and respiratory specimens, such as bronchoalveolar lavage fluid and sputum (Chen et al., 2020). However, they can be time-consuming and have a low detection rate. In blood culture, the detection rate is only 0–14% even in patients with severe pulmonary infection (Afshar et al., 2009; Zhang et al., 2019). In addition, it is often impossible to detect atypical pathogens such as *Legionella, Mycoplasma*, and *Chlamydia*. The sputum collection by most patients is not standardized, and it is easily contaminated by oropharyngeal colonizing bacteria. Therefore, it is also difficult to determine whether the test results are clinically significant. On the other hand, the detection of pathogens by antigen and/or antibody immunology and PCR must be based on the genetic sequence of known pathogens, and unknown pathogens cannot be detected (Miao et al., 2018).

Metagenomic next generation sequencing (mNGS) is a method that can directly utilize the patient specimen for pan-nucleic acid detection; all nucleic acids in the specimen are extracted and sequenced in parallel, so as to obtain the sequence of host and microorganisms (Gu et al., 2019; Mitchell and Simner, 2019). In recent years, mNGS has provided a more efficient and accurate means for pathogen diagnosis and updated the diagnostic strategy for lower respiratory tract infections. This study aimed to review the diagnostic value of mNGS for lower respiratory tract infections, the impact of different sampling methods on its detection efficiency, and the current technical difficulties in the clinical application of mNGS.

## Application of mNGS in the Diagnosis of Infectious Diseases of the Lower Respiratory Tract

mNGS does not rely on traditional microbial culture, nor does it require specific amplification. It has been widely applied in the nervous system and eye infections in recent years (Langelier et al., 2018; Zinter et al., 2019; Huang et al., 2020). mNGS can be applied to a wide range of specimen types (sputum, throat swab, blood, alveolar lavage fluid, pleural fluid, cerebrospinal fluid, pus, tissue specimens, etc.), and it can directly detect nucleic acids in clinical samples without distinction and selectivity for high-throughput sequencing. Comparing and analyzing with the known microbial sequence database, and judging the types of microorganisms contained in the sample based on the sequence information, it can unbiasedly detect pathogenic microorganisms in clinical samples, including viruses, bacteria, fungi, and parasites (Zinter et al., 2019).

Huang et al.carried out mNGS detection in 240 patients with suspected pulmonary infection (Huang et al., 2020). The results showed that the positive rate of mNGS was 88.30% in patients with pulmonary infection, while the positive rate of the traditional pathogen detection method was 25.73% . In patients with non-pulmonary infection, the positive rate of mNGS was 18.84%, while the positive rate of traditional pathogen detection method was 11.59%. This study showed that the sensitivity of mNGS (88.30%) for pathogen detection was much higher than that of traditional detection methods (25.73%), while the specificity of mNGS (81.16%) was slightly lower than that of traditional detection methods (88.41%). The reason may be is that the traditional detection methods could not detect all pathogenic microorganisms, leading to a higher true-negative rate of non-pulmonary infections. Among the specimens of patients with lung infections that were negative for traditional pathogen detection, mNGS detected microorganisms related to human diseases in 94.49% of the specimens. mNGS could detect and identify multiple pathogens simultaneously. The above results demonstrate that mNGS has more advantages than traditional detection methods. Chen et al. showed that in the cases of lower respiratory tract infection, the detection rate of mNGS to pathogenic bacteria was 65.0%, significantly higher than that of culture method (20.0%) (Chen et al., 2020).

However, based on the unbiased and highly sensitive characteristics of mNGS, false positive results may appear in the test report. It is a challenge for clinicians to distinguish pathogens from normal microorganisms and environmental pollutants (Han et al., 2019). Failure to properly interpret the test report may lead to erroneous disease diagnosis (Salter et al., 2014; Wilson et al., 2018). Nucleic acid contamination may occur from specimen collection to specimen processing, and may also come from the environment. For example, lung tissue specimens may be contaminated during biopsy. In addition, the sample collection container may also become a source of contaminated nucleic acid. Strictly formulating sample collection guidelines and strictly complying with them are essential to reduce the risk of nucleic acid contamination during sample collection (Simner et al., 2018). Studies have shown that most of the reagents used for mNGS will also introduce foreign DNA during the sequencing process. This phenomenon is called “kit-ome”, which will seriously affect the sample results (Afshinnekoo et al., 2017). Therefore, it is important to set a template-free control in the mNGS analysis to determine the nucleic acid background of the reagents used for sequencing, which can help filter out contaminated background readings during the interpretation of the results (Simner et al., 2018; Breitwieser and Salzberg, 2020).

For some culture-positive samples, mNGS results may also be negative (false negative problem) (Thoendel et al., 2018; Duan et al., 2021). Some pathogens with hard cell walls, such as fungi, may reduce the efficiency of nucleic acid extraction (Han et al., 2019). In addition, when the pathogen load is lower than the detection limit of mNGS, the detection may be missed. Taking viruses as an example, the relative abundance of viruses in samples is a key factor in whether high-throughput sequencing technology can detect them (Hall et al., 2014). If the virus is not enriched by physical methods, the main sequence information obtained by high-throughput sequencing is derived from human tissue cells or bacterial genomes, so the virus can only be detected at a relatively high concentration (Daly et al., 2011). The pre-sequencing treatment of samples such as filtration (Conceição-Neto et al., 2015; Li et al., 2015), centrifugation (Conceição-Neto et al., 2015), and nucleic acid removal using nucleases (Allander et al., 2001; Cann et al., 2005) can enrich viruses and improve the sensitivity of viral metagenomic analyses in respiratory samples. For other types of pathogens, nucleic acid enrichment of microorganisms and nucleic acid removal of hosts are also very necessary (Oechslin et al., 2018).

At present, in the diagnosis of lower respiratory tract infections, most studies focus on the comparison of the positive rates of all pathogens detected by mNGS and traditional pathogen detection methods. However, there are few studies on the comparison of the positive rates of bacteria, viruses and fungi detected by mNGS and traditional pathogen detection methods respectively, as shown in **Table 1**.

**Table 1:** Detection rate of pathogens of pulmonary infection in mNGS *versus* traditional detection methods.

Pathogen	mNGS (%)	Traditional method (%)	Ratio (mNGS/Traditional method)	References
**bacteria**	42.11	17.54	2.40	(Huang et al., 2020)
	76.40	47.20	1.65	(Fang et al., 2020)
	75.00	13.80	5.40	(Chen et al., 2021)
	73.10	8.30	8.80	(Chen et al., 2020)
	85.70	42.8	2.00	(Wang et al., 2020)
	50.00	15.00	3.33	(Li et al., 2018)
	65.00	20.00	3.25	(Chen et al., 2020)
**viruses**	35.09	0	–	(Huang et al., 2020)
	53.80	41.00	1.30	(Chen et al., 2020)
	82.98	0	–	(Chen et al., 2021)
	88.30	0	–	(Han et al., 2019)
	41.60	0	–	(Fang et al., 2020)
	84.30	28.10	3.00	(Wang et al., 2020)
**fungi**	38.60	8.19	4.71	(Huang et al., 2020)
	92.96	19.72	4.71	(Han et al., 2019)
	16.80	10.00	1.68	(Chen et al., 2021)
	90.40	4.70	19.23	(Fang et al., 2020)
	45.00	35.00	1.29	(Li et al., 2018)
	71.40	4.80	14.88	(Wang et al., 2020)

### The Diagnostic Value of mNGS in Bacterial Detection

For lower respiratory tract infection, traditional bacterial detection methods, such as blood culture sputum staining and culture urine antigen detection for molecular microbiology diagnosis, such as PCR, have some shortcomings like low positive rate, long turn-around time, limited types of pathogen detected, etc. (Cilloniz et al., 2016). Blood bacteriological tests require the acquisition of specimens before antibiotic use, with high specificity but low sensitivity (20%) (Waterer and Wunderink, 2001; Mandell et al., 2007). Similarly, sputum specimens need to be obtained before empirical antibiotic treatment. In order to improve the accuracy of microbial diagnosis, it is very important to obtain a qualified sputum specimen, which is defined as less than 10 epithelial cells and more than 25 lymphocytes (Cilloniz et al., 2016). However, many patients’ sputum specimens are not standardized in clinical practice, leading to unqualified samples, which further affects the microbiological diagnosis. For urine antigen testing, antigens of *Legionella serogroup 1* and *Pneumococcus* are excreted from the kidneys and can be detected by this method, which is independent of the use of antimicrobial therapy. It has been mentioned in the literature that the sensitivity and specificity for pneumococcus detection is 50% to 80% and 70% to 90%, and for *Legionella serogroup 1*, the sensitivity is 70% to 90% and the specificity is 99% (Cilloniz et al., 2016). Molecular microbiological diagnosis is increasingly widely used in the diagnosis of bacterial infection of the lower respiratory tract (Wellinghausen et al., 2009; Schulte et al., 2014; Mok et al., 2016). A study has shown that in the patients who received antibiotic treatment 72 hours before admission, the positive rate of bacterial pathogens detected by PCR was 78%, while the positive rate of culture method was only 32%, indicating that the effect of experiential antibiotic treatment on the detection rate of PCR was less than that of culture method (Gadsby et al., 2016).

Studies have shown that mNGS still has advantages over the culture method for bacterial detection (Wang et al., 2020). Henan Li et al. found that *Enterococcus faecalis* was detected by mNGS in lung tissue specimens of one lung cancer patient which was not found in the culture (Li et al., 2018). Since *Enterococcus faecalis* is not common in lung infections of immunocompetent patients, it was not considered a pathogen. Another case was a 6-year-old child who had been diagnosed with acute lymphoblastic leukemia and central nervous system disease. The culture was positive for *Penicillium*, which was not detected by mNGS. However, among the bacteria, *Neisseria meningitidis* was the most abundant. Although pulmonary infection caused by *Neisseria meningitidis* is uncommon, clinicians should still consider it as a possible pathogen of lung infection in immunodeficient patients. Therefore, clinicians need to correlate clinically to make accurate judgments when interpreting reports. Fang et al. (2020) found that for bacterial detection, the sensitivity and positive predictive value of mNGS were as high as 97.1% and 94.1%, respectively. Both are significantly higher than the traditional detection values. Traditional detection methods included bacterial and fungal smear and culture, Grocott’s methenamine staining, acid-fast staining, and blood sampling to detect routine blood and inflammatory markers in their study.

*Mycobacterium tuberculosis* is a special class of bacteria that we will discuss separately here. In 2018, only 55% of global tuberculosis cases were confirmed bacteriologically, while up to 60% of cases in China were bacteriologically negative. The tuberculosis diagnostic methods used in China (such as traditional acid-fast bacilli smear microscopy) may miss many tuberculosis-positive patients, resulting in a high proportion of false-negative patients (Perkins, 2006). Most tuberculosis tests in China, especially in rural areas, only use sputum smears due to the lack of culture, nucleic acid testing, etc. This will lead to delays in tuberculosis (TB) diagnosis and treatment, and continuous TB transmission (Yang et al., 2015).

At present, few studies have directly evaluated the value of mNGS in the diagnosis of TB infection. The culture method takes approximately 2-8 weeks, and only detects live bacilli. Furthermore, the history of anti-TB treatment has a significant impact on its sensitivity (Che et al., 2017). mNGS and gene Xpert detect nucleic acids rather than live bacilli, which are not affected by anti-TB drug treatment if done within 3 months. However, the sensitivity of both decrease significantly after more than 3 months of anti-TB treatment, possibly due to the undetectable tuberculous bacterial load after long-term treatment (Zhou et al., 2019). Liu et al. showed that the sensitivities of mNGS, Xpert, culture, and smear for detecting tuberculosis were 59.9%, 69.0%, 59.9%, and 24.6%, respectively (Liu et al., 2020). Before anti-tuberculosis treatment, the combined application of mNGS and conventional methods can increase the detection rate to 79.6% since mNGS is more advantageous in detecting mixed infections. Zhou et al. showed that in the identification of active TB cases, the sensitivity of mNGS was 44%, similar to that of the TB gene Xpert (42%), but significantly higher than that of traditional detection methods (29%) (Zhou et al., 2019). Due to the intracellular growth characteristics of tuberculosis, less nucleic acid is released to the outside of the cell, leading to difficult detection (Doughty et al., 2014). The combination of mNGS and Xpert, can help reach the diagnostic rate of 60%. According to the study, all three tests were significantly affected by anti-TB treatment since the sensitivity of pre-treatment samples was significantly higher than post-treatment samples. This suggests the importance of collecting clinical samples before empirical anti-TB treatment. In terms of time from clinical sample collection to report issuance, the gene Xpert can be released within 24 hours, while mNGS is released within 72 hours. Although it is longer than the former, it still has obvious advantages compared with the culture method. In terms of the amount sample requirements, mNGS requires an amount of about 1 ml, while the gene Xpert has an average detection dosage of 3.4 ml for cerebrospinal fluid samples and 80 ml for pleural effusion and ascites. This indicates that mNGS is a better choice for samples with relatively small amount of liquid.

### The Diagnostic Value of mNGS in Viral Detection

The traditional virus detection methods include antigen detection test, virus isolation, serological test, and nucleic acid amplification test. Antigen tests, such as rapid immunoassays and direct fluorescent antibody (DFA) Tests, have their own strengths and weaknesses (Loeffelholz and Chonmaitree, 2010). Rapid immunoassays provide results in less than 30 minutes and are widely used in the detection of respiratory virus, but their sensitivity is relatively low. Studies have shown that DFA is more sensitive than rapid immunoassays in virus detection, but inferior to virus isolation and/or reverse transcription-PCR (RT-PCR) (Vinh et al., 2008; Ginocchio et al., 2009). Virus isolation has long been considered as the gold standard for detecting respiratory viruses, with high sensitivity, but has long turn-around time in cell culture (Shetty et al., 2003). Serological diagnosis of respiratory viruses usually requires the collection of acute and convalescent sera to identify seroconversion or a fourfold increase in antibody titers. Therefore, testing for IgG is usually of little significance for the clinical management of patients. Testing for IgM antibodies can detect acute infection, but due to repeated exposure to the vaccine or circulating virus, serum IgM levels are often low, leading to reduced sensitivity to detection (Loeffelholz and Chonmaitree, 2010). Nucleic acid amplification tests, such as RT-PCR and nucleic acid sequence-based amplification (NASBA), are also commonly used for the detection of respiratory viruses with high sensitivity and specificity. Moreover, due to its high sensitivity, positive test results can still be obtained even if the patient has no symptoms (Jartti et al., 2008; van der Zalm et al., 2009), suggesting the persistence of nucleic acid after viral infection (Jartti et al., 2004; Kalu et al., 2010). However, the primers and probes need to be selected reasonably, and the virus species that can be detected is limited.

There have been some reports of mNGS for the detection of lower respiratory tract virus infection. The results of Huang et al. (2020) showed that among 171 patients with pulmonary infection, the positive results of mNGS were at 88.30% (151/171). The most commonly detected viruses were Human herpesvirus type 5 (HHV-5, n=26, 43.33%), Human herpesvirus type 4 (HHV-4, n=19, 31.67%), Human parvovirus B19 (HPV B19, n=4, 6.67%), and Torque teno virus (TTV, n=4, 6.67%), whereas the traditional detection methods were almost negative for all viruses. In another study of 72 patients with pulmonary infection, 30 cases had positive results on mNGS, while all of them were negative in traditional virus detection (Fang et al., 2020). Wang et al. (2020) showed that in 32 cases of children with severe pneumonia, two cases tested negative by bacterial culture, *M. pneumoniae* PCR, and D3 Ultra DFA Respiratory Virus Screening. mNGS detection confirmed Cytomegalovirus (CMV) and Bocavirus infection, respectively. Patients with CMV detection were finally diagnosed with leukemia, and antibiotic treatment was ineffective. Clinical symptoms (fever and shortness of breath) were quickly relieved after using antiviral drugs (ganciclovir). This indicates that CMV is not simply a endogenous virus, but is likely to be pathogenic. The population is generally susceptible to CMV, namely HHV-5. Like other herpes viruses, lifelong latent infection is established after the primary infection and can be periodically reactivated with the shedding of the infectious virus (Cannon et al., 2010), which can lead to the disease in people with low immunity and neonates (Dasari et al., 2013). Cytomegalovirus pneumonia is uncommon, and its diagnosis is somewhat challenging due to its lack of obvious specificity in clinical manifestations and imaging features (Fei et al., 2009; Skalski and Limper, 2016). There was a case report of a patient infected with Human Immunodeficiency Virus (HIV). Traditional pathogen tests, including specific antigen or antibody tests, PCR amplification and culture methods, did not find a clear pathogen. Finally, mNGS test confirmed the co-infection of *Pneumocystis jirovecii* and Cytomegalovirus, and the patient was cured and discharged after targeted treatment (Xie et al., 2021).

Although mNGS has some advantages in virus detection, there are still some problems to be solved. First of all, the extraction of viral nucleic acid is a key step in sample preparation, and it is difficult to detect new viruses resistant to extraction with the current mNGS technology. Secondly, the detected virus sequence does not necessarily indicate the presence of replicable viruses in the sample, and how to determine whether there is a full-length viral genome or whether there are infectious virus particles in the sample may require more research (Ng et al., 2014). In some cases, viral infection may be limited to a specific area of a tissue or part of susceptible cells, so the detection of a single or very few viral sequences may also indicate viral infection. Therefore, how to set the appropriate determination value of the number of viral sequences remains questionable (Cantalupo and Pipas, 2019). In addition, some studies have reported the false positive problem of mNGS in the detection of virus infection due to contaminants in the laboratory or reagents (Yozwiak et al., 2012; Naccache et al., 2013; Salter et al., 2014). Therefore, removal of contaminants is very important for accurately identifying whether there is virus in the sample.

### The Diagnostic Value of mNGS in Fungal Detection

For fungal pulmonary infection, traditional detection methods mainly include culture, histopathology, antigen detection, serology, imaging, and molecular diagnostics (Smith and Kauffman, 2012; Salzer et al., 2018). Culture method is often regarded as the gold standard for fungal infection, but most fungi are cultured for a long time, some for up to 1 month (Salzer et al., 2018). Histopathological diagnosis requires invasive examination, and its sensitivity and specificity are largely dependent on the pathologist’s experience, with a certain misdiagnosis rate (Bialek et al., 2002). For patients with normal immune function, the sensitivity of antigen detection may be low, on the contrary, the sensitivity of antibody detection is higher (Hage et al., 2011), while for immunosuppressed patients and patients in the acute course of disease, antibody detection may appear false negative (Salzer et al., 2018). In addition, for some mycosis diagnoses, such as paracoccidioidomycosis, serological examination is not required in most cases, but it can be used as a tool to monitor the treatment outcome (Vidal et al., 2014). The role of PCR in fungal detection is not yet clear and has not yet been standardized (Supparatpinyo et al., 1994).

In fungal infections, mNGS can detect pathogens that are not clear by traditional detection methods. This can help increase the detection rate of different fungi, thereby improving the accuracy of anti-infective treatment. However, it also has some limitations.

Li et al. showed that for patients with fungal pulmonary infection, the positive rate of mNGS detection was 92.96%, while the traditional pathogenic detection method was only 19.72% (Li et al., 2018). The combined analysis of mNGS and smear can shorten the detection time, provide more accurate bacterial identification, and can be used as a routine diagnostic tool for invasive fungal infections. Wang et al. found that mNGS had a significant effect on the detection of fungi (Wang et al., 2020). Among the 21 cases of fungal pneumonia, only 1 case was detected by culture, while the other 19 cases were detected by mNGS. The two cases missed by mNGS included one with cryptococcal pneumonia and one with Aspergillus pneumonia. In the latter, both the culture and galactomannan(GM) test results were positive. The above results indicate that although the positive rate of culture is low, the combination of culture, GM test, and mNGS is of great significance in the diagnosis of fungal pneumonia to avoid missing positive samples. It is worth mentioning that mNGS has no obvious diagnostic advantage for cryptococcal pneumonia. Among the four cases of cryptococcal pneumonia, the capsular polysaccharide antigen detection was positive for all. However, mNGS missed one case. Rapid on-site cytological evaluation(ROSE) also played an important role in the diagnosis of cryptococcal pneumonia. Granulomas were found in the transbronchial lung biopsy(TBLB) specimens of four patients, and *Cryptococcus* was found in 3 of 4 TBLB specimens. Therefore, for cryptococcal pneumonia, capsular polysaccharide antigen detection is recommended. mNGS is not necessary if the patient’s medical history, clinical manifestations, imaging manifestations, and ROSE results are highly suspected of cryptococcal pneumonia. The reasons are considered to be related to the low efficiency of nucleic acid extraction due to the hardness and insolubility of the fungal cell wall. However, some studies have shown that mNGS has no obvious advantages in fungal detection compared with traditional detection methods (Xie et al., 2019; Fang et al., 2020), and the two detection methods have their own advantages in the identification of specific fungi. For example, traditional detection methods have a higher detection rate for *Candida albicans* and *Candida tropicalis*, whereas mNGS has a higher detection rate for *Aspergillus* (Fang et al., 2020). Based on the above research results, clinicians should also consider the results of traditional methods when selecting mNGS.

### The Diagnostic Value of mNGS for Mixed Infection

A mixed infection is defined when two or more infectious pathogens are detected. For immunocompromised patients, such as patients receiving long-term or large amounts of steroids or other immunosuppressive drugs, solid organ transplant recipients, patients with hematologic malignancies, etc. (Azoulay et al., 2020), the risk of mixed infection is high.

Wang et al. conducted a retrospective study on 55 cases of pulmonary infection and confirmed that mNGS plays a prominent role in the diagnosis of mixed pulmonary infection (Wang et al., 2019). Therefore, mNGS is undoubtedly a more useful diagnostic tool for immunodeficient patients susceptible to infection by various pathogens. This conclusion was consistent with the study of Fang et al. (2020). The study also found that 55.6% of patients were diagnosed with mixed infection only by mNGS, while the diagnostic rate of mixed infection could be increased to 58.3% when combined with traditional detection methods. The above studies suggest that mNGS is helpful for clinicians in identifying patients with pulmonary infection, especially those with mixed infection in severe patients, and in guiding further treatment. However, mNGS technology cannot completely replace the commonly used clinical detection methods at present but can be used as an effective supplementary method for pathogen detection.

Li et al. (2018) detected a patient with severe pneumonia infected with Human rhinovirus C (HRV-C) and Human bocavir1 (HBoV1) by mNGS. Although antiviral treatment effectively alleviated the patient’s symptoms, it is difficult to determine which virus was the main causative agent. This situation also appears in the study of Zhou et al. (2016) in which mixed infection was confirmed by other methods, but it was not possible to distinguish which pathogen caused the manifestations of the disease. The possible explanation for this is that the immune profiles of patients with mixed infection vary from person to person, and the pathogens may vary in toxicity among different patients (Crockett and Keeffe, 2005). Therefore, clinicians need to accurately grasp the pathogenic mechanism of different pathogens in order to make appropriate clinical diagnosis and rational drug use.

## Influence of Different Sampling Methods on the Detection Efficiency of mNGS

In theory, mNGS can detect pathogens in clinical samples without bias and can distinguish pathogenic microorganisms from background microorganisms (Dietel et al., 2015). However, relatively few studies have been conducted on the selection of samples for mNGS detection of pulmonary infections.

Xu Chen et al. showed that the sensitivity of bronchoalveolar lavage fluid (BALF) mNGS samples (81.3%) was significantly higher than that of blood mNGS samples (25.0%) in detecting bacterial and fungal etiology (Chen et al., 2020). On the other hand, there was no significant difference in the specificity between the two. For virus detection, 19 patients had positive blood mNGS viral test results, while 12 patients had positive BALF mNGS viral test results. It is worth noting that blood mNGS detected *Epstein-Barr virus* (EBV) in nine patients, but the BALF mNGS of these nine patients were all negative. This result is consistent with the fact that there are fewer reports of pneumonia caused by EBV infection (McManus et al., 2009). This also suggests that EBV detected only by blood mNGS may not be related to pulmonary infection, but is more likely to be considered as a complication of host immune damage (Chen et al., 2020). The blood or BALF mNGS test results were positive for CMV in eight patients. The higher CMV sequence number detected in blood mNGS may be explained by the fact that the detected virus is not the pathogen that causes pulmonary infection, since some viruses may enter the blood from other parts of the body rather than the lung or may reactivate from the blood (Worth et al., 2016; Leng et al., 2017). In addition, EBV, also known as HHV-4, belongs to the same group of human herpesviruses as CMV, and causes latent infection (where the virus persists) and present infection. The virus mainly infects B lymphocytes, which may be why it was detected in blood samples rather than respiratory samples (Cherry-Peppers et al., 2003; Maeda et al., 2009). In general, BALF mNGS was more optimal for detection of bacteria and fungi than blood mNGS. However, blood mNGS was more sensitive in detecting viruses, but it has limited significance in lower respiratory tract infections.

Wang et al. used mNGS to detect pathogens in 39 patients with suspected peripheral pulmonary infection, and compared the microbial composition and diagnostic accuracy detected by three sampling methods: BALF, bronchial needle brushing (BB), and TBLB (Wang et al., 2020). The study found that several mNGS sampling methods of TBLB, BALF, BB, TBLB+BALF+BB have no statistically significant differences in sensitivity to bacterial infections. The same was true for fungi and unclassified lung pathogens. However, TBLB+BALF +BB had the highest sensitivity, followed by BB, BALF, and TBLB. The TBLB had the highest specificity, followed by BB and BALF, and there was no significant difference in the relative abundance of bacteria, fungi, and viruses in the three samples. A comprehensive consideration of sensitivity and specificity for mNGS detection showed that BB samples were the best.

Liu et al. combined ROSE, virtual bronchoscopic navigation, and radial probe endobronchial ultrasound (R-EBUS), using transbronchial lung biopsy (TBLB) and bronchoalveolar lavage (BAL) to collect specimens from patients with peripheral lung infections (Liu et al., 2019). They detected pathogenic microorganisms using mNGS, and compared the two sampling methods. The results showed that mNGS in the TBLB group had higher specificity, whereas mNGS in the BALF group had higher sensitivity. It is worth noting that BALF mNGS detects a wider range of pathogenic microorganisms and can narrow the range of suspected pathogens. On the other hand, the TBLB mNGS provides more accurate diagnostic results, while the hard-to-obtain specimens should be diagnosed using BALF sampling methods. A study showed that the positive rate of TBLB mNGS guided by R-EBUS (78.7%) was significantly higher than that of the TBLB group (60.0%), because R-EBUS significantly improved the accuracy of bronchoscopy in obtaining lesion specimens (Li et al., 2020). ROSE is a rapid cytological evaluation technology that can be used for tissue sample collection (Ganc et al., 2015). ROSE combined with mNGS technology can improve the accuracy of infection diagnosis, provide a more comprehensive information for subsequent treatment, reduce the use of antibiotics and improve the prognosis of patients (Liu et al., 2019).

At present, the number of studies regarding selection of samples for the detection of pulmonary infection by mNGS is relatively small, and most of the studies are single-center studies. At the same time, there is also lack of data in specific populations, such as immune dysfunction or immunosuppressed patients. More research is needed to address the recommended timing of the detection of mNGS for patients and the combination of assistive technologies.

### Technical Difficulties in Clinical Application of mNGS

As a rapidly emerging field, mNGS has advantages and limitations.

First is the high sensitivity of mNGS. mNGS can theoretically report all pathogens with known genome sequences. Currently, there are more than 8,000 known pathogens with high detection sensitivity. However, this also means that microbial contamination in the environment, reagents, containers, and colonizing microorganisms in the human body will affect the large number of non-pathogenic microorganisms contained in the report (false positive problem), and the pathogens are hidden in the colonization and background microorganisms (Salter et al., 2014; Wilson et al., 2018).

Next, the detection rate of intracellular bacteria and pathogens with hard cell walls is low. Due to the characteristics of intracellular growth, the content of nucleic acids released into extracellular body fluids by intracellular bacteria is low, while for some pathogens with hard cell walls, such as fungi, the extraction efficiency of nucleic acid is low, leading to low detection sensitivity of both (Han et al., 2019; Zhou et al., 2019). Therefore, even if the sequence number of these types of pathogens in the detection report is not high, the possibility that they are pathogenic should still be considered.

Third, the RNA detection is difficult to perform. The abundance of RNA is positively correlated with the degree of gene transcription activity. Detection of RNA can identify dead and live bacteria, and distinguish between current and past infections. Therefore, compared with DNA sequencing alone, DNA and RNA sequencing have multiple advantages. However, compared to DNA, human-derived RNA has higher abundance and complexity, and is easily degraded, which brings higher requirements for sample transportation and storage.

Fourth, is the cost of mNGS, which is relatively high. One study mentioned that the cost of mNGS testing with blood, cerebrospinal fluid, and respiratory samples ranges from US $1000 to US $2500 (Ramachandran and Wilson, 2020). Another study noted that in-house platform sequencing requires significant resources for equipment and reagents, which can cost hundreds of thousands of dollars; the average cost per test ranges from US $2000 to US $3500 for outsourced sequencing (Mitchell and Simner, 2019). Ninety percent of reads detected by mNGS were host-derived (Salzberg et al., 2016; Simner et al., 2018), so most of the cost of detection was spent on invalid sequences. Therefore, host nucleic acid depletion methods applied before sequencing can effectively reduce the cost of generation and analysis of redundant data. It has been shown that if multiple samples can be sequenced at a time, the cost of each sample can be significantly reduced, but the detection speed will be sacrificed (Rutanga et al., 2018; Han et al., 2019).

Fifth, mNGS cannot yet fully detect drug resistance. mNGS can sequence the pathogen genome, so it has some advantages in the detection of antibiotic resistance genes (ARGs) (Graf et al., 2016), and it has been confirmed by some studies (Andersen et al., 2016; Grumaz et al., 2016; Ruppé et al., 2017). Third-generation sequencing technology (Oxford Nanopore sequencing and PacBio SMRT sequencing) has a greater advantage in detecting ARGs (Schmidt et al., 2017). Even so, there is still a high error rate, which is estimated to be as high as 10%-15% (Goldstein et al., 2019; Vasudevan et al., 2020). Moreover, there is a certain gap in the degree of association between drug resistance genotypes and phenotypes reported so far. The analysis of ARGs requires extremely high sequencing depth and would cost thousands times more. At present, some drug resistance gene databases have been developed (Liu and Pop, 2009; Zankari et al., 2012; Jia et al., 2017; Lakin et al., 2017; Arango-Argoty et al., 2018), but all of them have certain limitations and cannot be used for clinical diagnosis. For example, CARD database is limited to identify known ARGs, and ignores the heterogeneity of resistance mechanisms, such as point mutations, gene expression changes, and post-translational modifications (Grumaz et al., 2016).

Sixth, is long turnaround time for mNGS. Depending on the sequencing technology, methods, and bioinformatics procedures, the turnaround time of mNGS is about 6 hours to 1 week from the time the sample is received, with an average of 48 hours (Wilson et al., 2014; Greninger et al., 2015; Pendleton et al., 2017).

Lastly, interpretation of mNGS results is a huge challenge. Due to contamination problems, sample extraction problems, host immune response and other factors, the mNGS test results are very complicated (Ganc et al., 2015; Hart et al., 2015). Some institutions have established precision medicine teams composed of representatives of medical microbiology, infectious disease, computational biology, and other clinician groups to discuss mNGS results and provide reasonable explanations (Mongkolrattanothai et al., 2017).

In summary, mNGS has some advantages over traditional methods in detecting pathogens of lower respiratory tract infection. At the same time, mNGS has diagnostic value for bacteria, fungi, viruses, and mixed infections. It can detect pathogens that are not clear in traditional detection methods and improve the accuracy of anti-infective treatment. However, due to the problems of false positive, low detection rate of some pathogens and high cost, clinicians should also consider the results of traditional culture when selecting mNGS and make accurate judgments based on clinical practice.

In terms of tuberculosis diagnosis, mNGS has the same diagnostic efficiency with the gene Xpert. mNGS combined with traditional tuberculosis detection methods can significantly increase the detection rate of pathogens while anti-tuberculosis treatment for more than 3 months will significantly reduce the diagnostic efficiency. Collecting clinical samples before empirical anti-TB treatment is of great significance. mNGS testing requires a smaller sample size, which is more suitable for testing samples with a small amount of liquid.

In terms of mNGS sample selection, the TBLB, BALF, BB, TBLB+BALF+BB sampling methods showed no statistically significant differences in the sensitivity of detection of bacterial infections. The same is true for fungi and unclassified lung pathogens. However, the TBLB +BALF+BB had the highest sensitivity, followed by BB, BALF, and TBLB. TBLB had the highest specificity, followed by the BB and BALF. There was no significant difference in the relative abundance of pathogens, fungi, and viruses among the three samples. mNGS is effective for the detection of fungi. It has certain advantages in the detection of mixed lung infections in immunodeficient patients, but it has certain limitations in the diagnosis of certain types of lung infections (such as cryptococcal pneumonia).

mNGS is a technology with obvious advantages; however, it also has drawbacks. Therefore, with reasonable standardization for clinical users with clinical indications, reasonable selection of clinical samples, in combination with the experiences of clinicians, this technology will surely become a powerful tool for the precise detection of infectious diseases and targeted drug treatment.

## Author Contributions

YZ wrote the main part of the manuscript. TW, XQ, and JZ revised the manuscript. All authors contributed to the article and approved the submitted version.

## Funding

This work was supported by Beijing Municipal Key Clinical Specialty Project (grant no.2020-129).

## Conflict of Interest

The authors declare that the research was conducted in the absence of any commercial or financial relationships that could be construed as a potential conflict of interest.

## Publisher’s Note

All claims expressed in this article are solely those of the authors and do not necessarily represent those of their affiliated organizations, or those of the publisher, the editors and the reviewers. Any product that may be evaluated in this article, or claim that may be made by its manufacturer, is not guaranteed or endorsed by the publisher.
